# Supplementary material for: Effects of Alberta Family Integrated Care (FICare) on Preterm Infant Development: Two Studies at 2 Months and between 6 and 24 Months Corrected Age
Source: J Clin Med. 2022 Mar 18;11(6):1684. doi: 10.3390/jcm11061684 (PMC8952230; doi:10.3390/jcm11061684)
Supplement: Supplementary file 1 [file jcm-11-01684-s001.zip › jcm-1631416-supplementary.pdf]

Supplementary Table S1. Study 1 hierarchical regression models of variables associated with risk of delay in each developmental domain at 2 months corrected age.

Variable		Odds Ratio	95% Confidence Interval	p-value
**Communication Domain**				
Group	Standard care	1.00		
	Alberta FICare™	0.13	0.00–4.12	0.25
Hospital type	Regional	1.00		
	Urban	0.11	0.00–7.62	0.31
Singleton/twin	Singleton	1.00		
	Twin	1.02	0.02–51.86	0.99
Income	< $80,000	1.00		
	≥ $80,000	0.42	0.01–18.39	0.65
	Prefer not to answer/ don't know	0.35	0.00–77.63	0.70
Mother's age		0.91	0.66–1.26	0.57
STAI Trait Anxiety [a]		0.97	0.80–1.17	0.75
PSI-4-SF Parental Distress		0.94	0.72–1.24	0.68
PSI-4-SF Dysfunctional Interaction		1.38	0.84–2.28	0.20
PSI-4-SF Difficult Child		1.02	0.66–1.56	0.93
**Problem Solving Domain**				
Group	Standard care	1.00		
	Alberta FICare™	2.57	0.13–49.32	0.53
Birth hospitalization length of stay		1.12	0.97–1.30	0.12
Education		0.68	0.11–3.99	0.67
EPDS		1.07	0.58–1.99	0.83
STAI State Anxiety		0.95	0.71–1.27	0.71
STAI Trait Anxiety [a]		1.00	0.83–1.21	0.99
PSI-4-SF Parental Distress		1.00	0.77–1.29	0.98
PSI-4-SF Dysfunctional Interaction		1.16	0.76–1.77	0.50
PSI-4-SF Difficult Child		0.93	0.64–1.36	0.72
**Personal-Social Domain**				
Group	Standard care	1.00		
	Alberta FICare™	0.69	0.26–1.85	0.47
Mother's age		0.92	0.82–1.02	0.11
EPDS		0.94	0.77–1.15	0.57
STAI State Anxiety		1.06	0.97–1.17	0.21
STAI Trait Anxiety [a]		1.03	0.96–1.10	0.45
PSI-4-SF Parental Distress		0.99	0.91–1.08	0.80
PSI-4-SF Dysfunctional Interaction		1.11	0.95–1.29	0.19
PSI-4-SF Difficult Child		1.01	0.90–1.14	0.84

Abbreviations: Alberta FICare™, Alberta Family Integrated Care™; STAI, State-Trait Anxiety Inventory; PSI-4-SF, Parenting Stress Index, Fourth Edition Short Form; EPDS, Edinburgh Postnatal Depression Scale. Note: Bold used to delineate categories for measurement scales. [a] measured only at admission to NICU.

Supplementary Table S2. Study 2 hierarchical regression models of variables associated with risk of delay in each developmental domain between 6 and 24 months corrected age.

**Variable**		**Odds Ratio**	**95% Confidence Interval**	**p-value**
**Communication Domain**				
Group	Standard care	1.00		
	Alberta FICare™	0.09	0.01-0.62	0.01
Mother born in Canada	No	1.00		
	Yes	0.17	0.04-0.76	0.02
Mother’s age		1.00	0.96-1.40	0.13
**Problem Solving Domain**				
Group	Standard care	1.00		
	Alberta FICare™	0.10	0.00-6.04	0.27
STAI Trait Anxiety [a]		1.14	0.93-1.41	0.21
PSI-4-SF Dysfunctional Interaction		1.21	0.86-1.70	0.27
**Personal-Social Domain**				
Group	Standard care	1.00		
	Alberta FICare™	0.77	0.06-9.16	0.84
Singleton/twin	Singleton	1.00		
	Twin	5.22e+08	-	0.997
Income	< $80,000	1.00		
	≥ $80,000	0.12	0.01-1.41	0.09
	Prefer not to answer/ don’t know	1.11e-08	-	0.99
Employment		23.40	1.00-545.50	0.05
Birth weight		1.00	1.00-1.01	0.08
STAI Trait Anxiety [a]		1.03	.90-1.18	0.69
PSI-4-SF Difficult Child		1.44	1.01-2.05	0.04

Abbreviations: Alberta FICare, Family Integrated Care™; STAI, State-Trait Anxiety Inventory; PSI-4-SF, Parenting Stress Index, Fourth Edition Short Form. Note: Bold used to delineate categories for measurement scales. [a] measured only at admission to NICU.
